# Potent activity of prostaglandin J_2_ on prostanoid DP receptors

**DOI:** 10.1016/j.jbc.2025.108523

**Published:** 2025-04-18

**Authors:** Kanaho Senoo, Keijo Fukushima, Hitomi Yamamoto, Ayaka Hamaguchi, Akiko Suganami, Harumi Takano, Mayu Yamashita, John W. Regan, Yutaka Tamura, Hiromichi Fujino

**Affiliations:** 1 Department of Pharmacology for Life Sciences, Graduate School of Pharmaceutical Sciences & Graduate School of Biomedical Sciences, Tokushima University, Tokushima, Japan; 2 Department of Bioinformatics, Graduate School of Medicine, Chiba University, Chiba, Japan; 3 Department of Pharmacology and Toxicology, College of Pharmacy, The University of Arizona, Tucson, Arizona, USA

**Keywords:** G-protein coupled receptors, receptor regulation, prostaglandin, cAMP, β-catenin, DP prostanoid receptors, PGD_2_, PGJ_2_, biased agonist, operational model calculation

## Abstract

Prostaglandin D_2_ (PGD_2_), an anti-inflammatory mediator, is acting through Gs-protein coupled D-type prostanoid (DP) receptors. DP receptors are not extensively distributed; in tissues, they are the least abundant among members of the prostanoid receptor family, whereas their primary ligand PGD_2_ is the main prostanoid in most tissues. PGD_2_ is dehydrated or isomerized to a number of metabolites enzymatically or nonenzymatically. To understand why many metabolites of PGD_2_ are produced *via* different pathways, regular cell-based experiments, Black/Leff operational model calculations, and *in silico* simulations were utilized. Here we show that, among the five metabolites of PGD_2_, prostaglandin J_2_ (PGJ_2_) was the most potent metabolite for DP receptors, particularly in the cAMP signaling pathway. This result was attributed to PGJ_2_ forming an extra and/or stronger hydrogen bond by more negatively charged carbonyl in the cyclopentene ring with DP receptors than PGD_2_. Therefore, when PGD_2_ is released into the blood, it would activate DP receptors, which are then continuously activated by PGJ_2_ to sustain the DP receptor/cAMP-mediated signaling pathway. Thus, the anti-inflammatory effects of PGD_2_ may be taken over/out competed and/or even enhanced by PGJ_2_. Here, PGJ_2_ was found to be a standout mediator of cAMP-mediated signaling pathway, which induces more potent and prolonged DP receptor activities as a biased ligand, possibly for resolving the inflammatory reaction. Moreover, since each metabolite showed different properties, these results provide insight into why many metabolites of PGD_2_ are produced and the miscellaneous physiological roles induced by the main prostanoid in most tissues through the least abundant DP receptors.

Prostaglandin D_2_ (PGD_2_) was initially identified in inflamed tissues and fluids; therefore, it was characterized as a proinflammatory mediator at that time (1, 2). However, PGD_2_ is now widely considered to play a role in both proinflammatory and anti-inflammatory mechanisms (3). These contradictory roles in inflammation may be partly because of the receptors that PGD_2_ binds to. In general, Gs-protein coupled D-type prostanoid (DP) receptors, also known as DP1 receptors, are involved in anti-inflammatory functions, whereas mainly Gi-protein coupled chemoattractant receptor homologous molecules expressed on Th2 cell (CRTH2) receptors, also known as DP2 receptors, play a role in proinflammatory functions (3).

CRTH2 receptors belong to a group of chemotactic G-protein coupled receptors, making them phylogenetically distant from other classical prostanoid receptor family members, including DP receptors (4, 5). Interestingly, DP receptors are not extensively distributed; in tissues, they are the least abundant among classical prostanoid receptor family members (4). Therefore, DP receptors have been suggested to play roles in some specific functions within relatively restricted areas, which have been studied and reported, particularly in central sleep regulation (4, 6) and peripheral anti-inflammatory mechanisms (4, 5, 6). Indeed, in general, the accumulation of cAMP is associated with inhibition of the activities of immune cells, such as dendritic and regulatory T cells (1, 4). In terms of cellular signaling pathways, besides Gs-protein–mediated cAMP/PKA signaling, DP receptors are known to activate T-cell factor (TCF)/β-catenin-mediated signaling, possibly *via* DP receptor–associated β-arrestin (7).

In contrast to the least abundant DP receptors, their primary ligand PGD_2_ is considered the major prostanoid in most tissues (8, 9). For example, PGD_2_ is the most abundant prostanoid in the brain (10, 11), whereas a large amount of PGD_2_ is also produced by mucosal mast cells (12, 13), and some is generated by enterocytes (14, 15). It is well known that this prostanoid is dehydrated or isomerized to a variety of metabolites enzymatically or nonenzymatically (3, 9, 11, 16), as presented in Figure 1. Thus, PGD_2_ is enzymatically metabolized to 13,14-dihydro-15-keto PGD_2_ (DK-PGD_2_) by the action of 15-hydroxyprostaglandin dehydrogenase pathway. PGD_2_ also spontaneously dehydrates to 15-deoxy-Δ^12,14^-PGD_2_ (15-d-PGD_2_) or prostaglandin J_2_ (PGJ_2_). 15-d-PGD_2_ or PGJ_2_ is further dehydrated to 15-deoxy-Δ^12,14^-PGJ_2_ (15-d-PGJ_2_). PGJ_2_ is also isomerlized to Δ^12^-PGJ_2_ *via* albumin-dependent mechanisms (3, 9, 11, 16). Moreover, Δ^12^-PGJ_2_ was reported to further dehydrate to 15-d-PGJ_2_ (3, 9, 16). Some of these metabolites were reported as ligands of peroxisome proliferator–activated receptors (PPAR) (11, 17, 18). For example, Δ^12^-PGJ_2_ and 15-d-PGJ_2_ were reported to promote neuroprotection *via* anti-inflammatory and/or antioxidant-dependent mechanisms (18). Conversely, J series of metabolized prostanoids of PGD_2_, such as PGJ_2_, Δ^12^-PGJ_2_, and 15-d-PGJ_2_, have electrophilic α,β-unsaturated carbonyl groups in their cyclopentenone rings, the carbons at the ninth position are considered electrophilic and chemically reactive, so that they can act as Michael addition acceptors (11, 17, 18). Moreover, Δ^12^-PGJ_2_ and 15-d-PGJ_2_ have additional reactive carbons at the 13th position of both prostanoids (11, 17, 18). These reactive carbons of J series prostanoids are able to form covalent bonds with cellular proteins that have free cysteine thiols including glutathione and induce receptor-independent physiological actions, such as neurodegeneration in the brain (11). Of note, the half-life of PGD_2_ has been reported to be 0.9 min in blood (19) and 30 min in plasma (16); however, its metabolites are considered more stable than their precursor, PGD_2_ (16).

**Figure 1:**
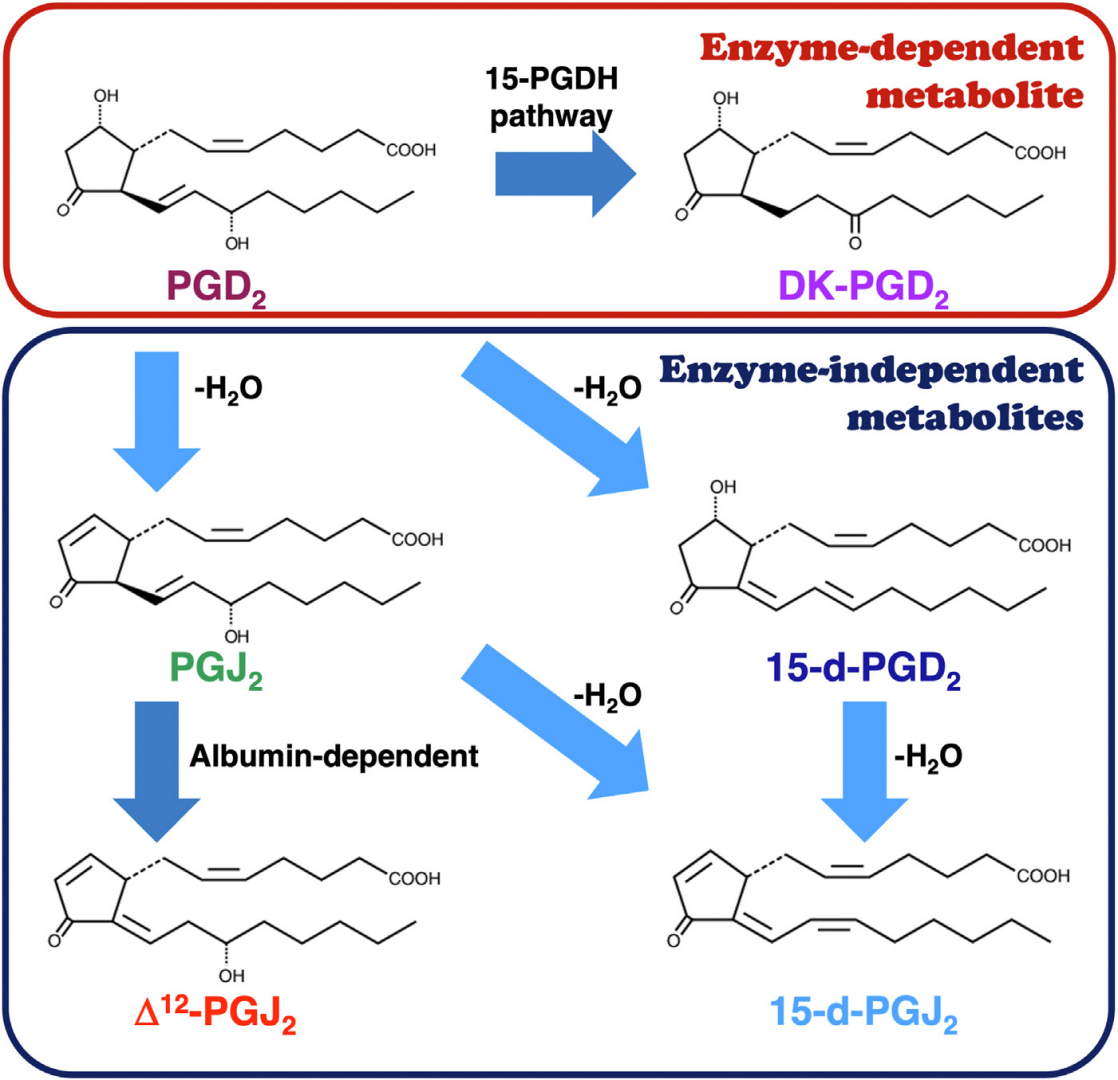
**Structures and metabolized pathways of PGD_2_ and its metabolites**. PGD_2_ and the enzyme-dependent metabolite is enclosed in a *red line*, whereas enzyme-independent metabolites are enclosed in a *blue line*. DK-PGD_2_, 13,14-dihydro-15-keto PGD_2_; P15-PGDH, 15-hydroxy prostaglandin dehydrogenase; 15-d-PGD_2_, 15-deoxy-D^12,14^-PGD_2_; 15-d-PGJ_2_, 15-deoxy-D^12,14^-PGJ_2_; PGD_2_, prostaglandin D_2_; PGJ_2_, prostaglandin J_2_.

We previously showed that the metabolite of prostaglandin E_2_ (PGE_2_), 15-keto-PGE_2,_ acted as a biased and switched agonist of E-type prostanoid (EP) 2 receptors from PGE_2_-activated EP4 receptors. Thus, 15-keto-PGE_2_ may not be an inactive metabolite of PGE_2_ but may mildly terminate and/or restore the PGE_2_/EP4 receptor–induced inflammatory reaction to maintain homeostasis of cells as an important mediator for resolution of inflammation through EP2 receptors (20). As we showed previously, human DP receptors and EP2 receptors are regarded as the most closely related receptors among the classical prostanoid receptor family, as duplicated gene products (5, 6). Therefore, it is possible to consider that some metabolites may have functions to take over/out compete and sustain signaling evoked by PGD_2_ as biased and/or switched agonists, since it is well known that PGD_2_ is readily dehydrated or isomerized to a variety of metabolites. However, difficulties are associated with monitoring/measuring the ratios of PGD_2_ and PGD_2_ metabolites as well as each ligand-induced signaling in regular cultured cell–based assay methods. Therefore first, we examined the effects of each prostanoid, PGD_2_ and its metabolites, on DP receptor–mediated signaling pathways in regular cell-based experiments. Then, in order to estimate the roles of these metabolites in PGD_2_-activated DP receptor–mediated functions, by the Black/Leff operational model calculations and *in silico* simulations. Here, we show that when PGD_2_ is metabolized to DK-PGD_2_ or the line of 15-d-PGD_2_ to 15-d-PGJ_2_, an extinction alliance, activities of DP receptors evoked by PGD_2_ would be promptly terminated. However, if PGD_2_ is metabolized to the line of PGJ_2_ to Δ^12^-PGJ_2_, a sustained activation alliance, activities of DP receptors evoked by PGD_2_ would be sustained or even enhanced. Thus, depending on the environment and which metabolic line would be activated, the physiological functions of initially PGD_2_-stimulated DP receptors would be markedly changed. These results provide insight into why so many metabolites of PGD_2_ are produced *via* different pathways and also the miscellaneous physiological roles induced by the main prostanoid in most tissues through the least abundant DP receptors.

## Results and discussion

**Figure 2:**
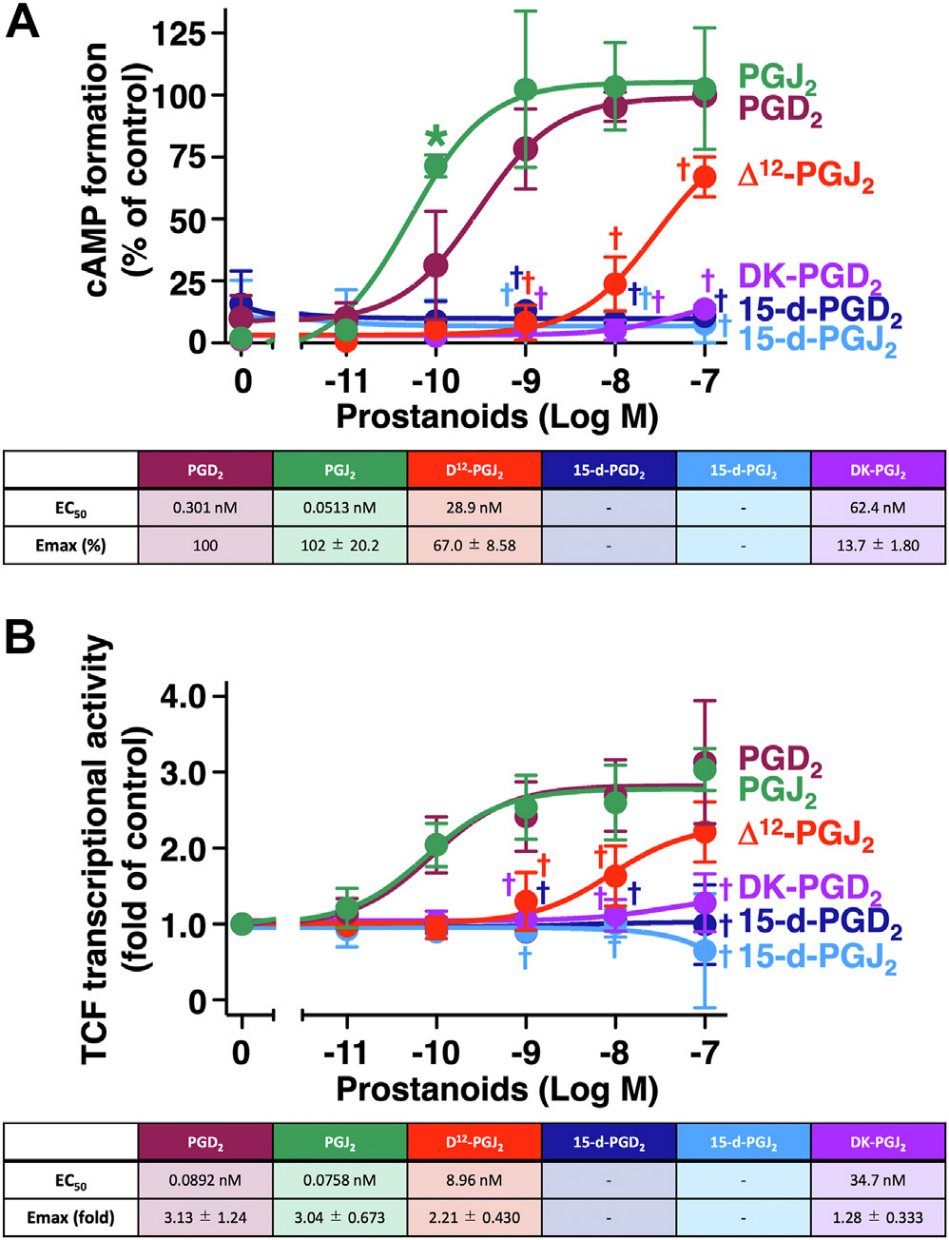
**Effects of PGD_2_ and its metabolites on cAMP formation and β-catenin/TCF-mediated luciferase transcriptional activities in HEK-DP cells**. HEK-DP cells were treated with vehicle or the indicated concentrations of PGD_2_ or its metabolites for 60 min for the cAMP assay (*A*) or for 16 h for the β-catenin/TCF-mediated luciferase assay (*B*). The table shows EC_50_ values and Emax values of PGD_2_- or its metabolite-stimulated formation of cAMP (*A*) or β-catenin/TCF-mediated luciferase activities in HEK-DP cells (*B*). Data are normalized to 100 nM PGD_2_-stimulated formation of cAMP as 100% and are the mean ± SD (error bars) (*A*) or to each vehicle-treated control as 1.0 and are the mean ± SD (error bars) (*B*), of at least three independent experiments, each performed in duplicate. ∗*p* < 0.05: analysis of variance significantly higher than the corresponding concentrations of PGD_2_. †*p* < 0.05: analysis of variance significantly lower than the corresponding concentrations of PGD_2_. DK-PGD_2_, 13,14-dihydro-15-keto PGD_2_; 15-d-PGD_2_, 15-deoxy-D^12,14^-PGD_2_; 15-d-PGJ_2_, 15-deoxy-D^12,14^-PGJ_2_; HEK-DP cells, human embryonic kidney-293 cell stably expressing human DP receptors; PGD_2_, prostaglandin D_2_; PGJ_2_, prostaglandin J_2_; TCF, T-cell factor.

To evaluate the agonistic effects of PGD_2_ and its metabolites, human embryonic kidney 293 (HEK-293) cells stably expressing human DP receptors (HEK-DP cells) were treated with indicated concentrations of PGD_2_ or its metabolites, DK-PGD_2_, 15-d-PGD_2_, PGJ_2_, Δ^12^-PGJ_2_, and 15-d-PGJ_2_, for 60 min, and then the cAMP formation assay was performed. As shown in Figure 2*A*, PGD_2_ led to a concentration-dependent increase in the formation of cAMP with EC_50_ of approximately 0.3 nM in HEK-DP cells, being consistent with our previous reports (7, 21). PGJ_2_ was also able to activate DP receptors as a full agonist so that it produced cAMP to Emax levels similar to those of PGD_2_. However, unexpectedly, the EC_50_ value of PGJ_2_ in HEK-DP cells was approximately 0.05 nM, being about 10 times lower than that of PGD_2_. The double-bond isomer of PGJ_2_, Δ^12^-PGJ_2_, showed smaller Emax and larger EC_50_ values, approximately 67% of the PGD_2_-evoked value and 28.9 nM, respectively, so that Δ^12^-PGJ_2_ may act as a partial agonist of DP receptors. In the case of DK-PGD_2_, this metabolite showed a partially agonistic effect with Emax and EC_50_ values of approximately 14% of the PGD_2_-evoked state and 62.4 nM, respectively. Conversely, the other metabolites, 15-d-PGD_2_ and 15-d-PGJ_2_, showed little effects on DP receptors in terms of cAMP accumulation, at least up to 100 nM.

As described earlier in the *Introduction* section, DP receptors have been reported to be able to stimulate TCF/β-catenin-mediated transcriptional activity (7). Therefore, the effects of PGD_2_ metabolites along with PGD_2_ on TCF/β-catenin activity were examined in HEK-DP cells. As shown in Figure 2*B*, HEK-DP cells treated with the indicated concentrations of PGD_2_ or PGJ_2_ for 16 h showed the activation of TCF/β-catenin activities to similar levels as full agonists, being approximately threefold, and EC_50_ values around 0.08 nM, whereas Δ^12^-PGJ_2_ led to approximately 70% of the Emax value of PGD_2_ (2.21-fold) with approximately 100 times higher EC_50_ values than those of PGD_2_ and PGJ_2_, at 8.96 nM. In the case of DK-PGD_2_, the Emax value was about 1.28-fold, being approximately 40% of the PGD_2_-evoked value, with an EC_50_ value of 34.7 nM. However, up to 100 nM, other metabolites, 15-d-PGD_2_ and 15-d-PGJ_2_, showed little effect on DP receptor–mediated activation of TCF/β-catenin signaling, similar to cAMP accumulation, as shown in Figure 2*A*. It is important to note that the physiological concentrations of prostanoids are approximately subnanomolar to 10 nM (5); therefore, the cAMP or TCF/β-catenin activity assays shown in Figure 2 were performed using up to 100 nM of PGD_2_ and its metabolites.

**Figure 3:**
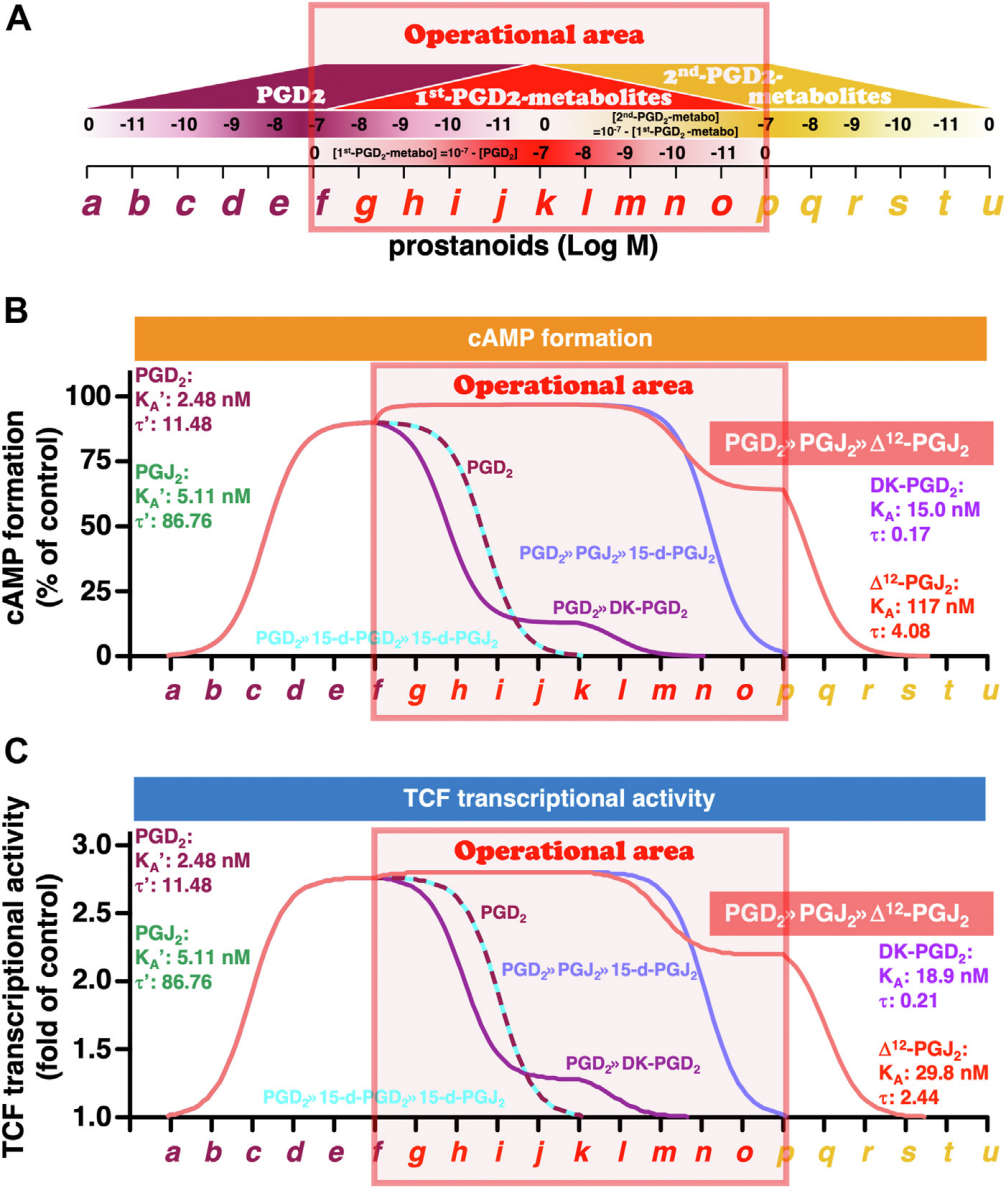
**The simulated total effects/responses of cAMP and TCF/β-catenin-mediated transcriptional activities of DP receptors evoked by PGD_2_ followed by 1st-PGD_2_-metabolites then 2nd-PGD_2_-metabolites**. *A*, schema of the increase or decrease of PGD_2_ and its 1st-PGD_2_-metabolites than 2nd-PGD_2_-metabolites, with the area in which PGD_2_, 1st-, or 2nd-PGD_2_-metabolites exist named the operational area, from 10^-7^ M PGD_2_ to each 10^-7^ M 2nd-PGD_2_-metabolite. Shown are the simulated total amounts of cAMP formation *B*, and TCF/β-catenin-mediated transcriptional activities (*C*) of DP receptors with PGD_2_ followed by its first and then second metabolites. *Dashed wine-red line*, PGD_2_ alone; *purple line*, PGD_2_ to DK-PGD_2_; *dashed sky-blue line*, PGD_2_ to 15-d-PGD_2_ then 15-d-PGJ_2_; *blue line*; PGD_2_ to PGJ_2_ then 15-d-PGJ_2_: *salmon-pink line*, PGD_2_ to PGJ_2_ then Δ^12^-PGJ_2_. The K_A_' values of PGD_2_ and PGJ_2_ used IC_50_ values of the binding assay shown in Figure 4*A*. The K_A_ values of metabolites are apparent affinity values determined by Black/Leff operational model calculation. The Tau (τ) values for partial agonists are logical/operational efficacies obtained using K_A_ values. The Tau' (τ′) values for full agonists were obtained using R and K_A_' values. DK-PGD_2_, 13,14-dihydro-15-keto PGD_2_; DP, D-type prostanoid; 15-d-PGD_2_, 15-deoxy-D^12,14^-PGD_2_; 15-d-PGJ_2_, 15-deoxy-D^12,14^-PGJ_2_; PGD_2_, prostaglandin D_2_; PGJ_2_, prostaglandin J_2_; TCF, T-cell factor.

As described earlier in the *Introduction* section, difficulties are associated with monitoring/measuring the ratios of PGD_2_ and PGD_2_ metabolites as well as each ligand-induced signaling in regular cultured cell–based assay methods. Therefore, to estimate the roles of DK-PGD_2_, 15-d-PGD_2_, PGJ_2_, Δ^12^-PGJ_2_, and 15-d-PGJ_2_ on DP receptors after PGD_2_ has been metabolized, next, the Black/Leff operational model was performed using data of Emax and EC_50_ values obtained, as presented in Figure 2, being similar to those we previously utilized (20, 22). The Black/Leff operational model adapt the fitting of experimental results, such as Emax values and EC_50_ values, to the occurrence of ligand-stimulated response cooperatively. Operational model calculations provided K_A_ values representing logical/operational ligand affinity and Tau (τ) values representing logical/operational ligand efficacy, and by utilizing and applying these parameters, the effect magnitude at each concentration of each ligand was calculated when two ligands were coexisting and stimulating the same receptor (23). Based on the results obtained in Figure 2, the best-fit curves of cAMP formation and TCF/β-catenin-mediated activity stimulated by PGD_2_ and its metabolites in HEK-DP cells were regressed. First, the regressed PGD_2_ concentration–response curve was plotted, and then the reverse style of the PGD_2_ concentration–response curve with the presence of 1st-PGD_2_-metabolite was plotted. When PGD_2_ reached a maximal concentration of 10^-7^ M (Fig. 3*A*,f), PGD_2_ was considered to be metabolized to 1st-PGD_2_-metabolites, either DK-PGD_2_, 15-d-PGD_2_, or PGJ_2_. Therefore, the concentration of PGD_2_ would decrease in reverse increments (Fig. 3, *B* and *C*, *dashed wine-red line*), and along with the decrease in the concentration of PGD_2_, its 1st-metabolites were increased. Secondarily, in the case of 15-d-PGD_2_ and PGJ_2_, when these 1st-metabolites reached a maximal concentration of 10^-7^ M (Fig. 3*A*,k), then they were considered to be metabolized to 2nd-PGD_2_-metabolites, either 15-d-PGJ_2_ or Δ^12^-PGJ_2_. Therefore, following reversely plotted 1st-metabolite curves, the reverse style of the 1st-PGD_2_-metabolite–concentration curve with the presence of 2nd-PGD_2_-metabolite was plotted. Again, the concentration of first metabolites would decrease in reverse increments with an increase in second metabolites. When first metabolites were completely metabolized to second metabolites, the maximal concentration of second metabolites, 10^-7^ M (Fig. 3*A*,p), would finally decrease in reverse increments to 0 nM (Fig. 3*A*,u). Thus, four PGD_2_-metabolic lines: PGD_2_>>DK-PGD_2_ (*purple line*), PGD_2_>>15-d-PGD_2_>>15-d-PGJ_2_ (*dashed sky-blue line*), PGD_2_>>PGJ_2_>>15-d-PGJ_2_ (*blue line*), and PGD_2_>>PGJ_2_>>Δ^12^-PGJ_2_ (*salmon-pink line*) of continuous cAMP formation (Fig. 3*B*) or continuous TCF/β-catenin-mediated activity (Fig. 3*C*) were plotted. As shown in Figure 3*B* in the operational area, if all PGD_2_s are metabolized to DK-PGD_2_, as plotted on the *purple line*, the formed cAMP would decrease faster than in the presence of PGD_2_ alone (*dashed red-wine line*), but the formation would be slightly prolonged until DK-PGD_2_ reduction to around 10^-9^ M (Fig. 3*A*,m). This is because DK-PGD_2_ would retain its affinity for DP receptors, although weak, and so this metabolite would compete with PGD_2_, so that the effect of PGD_2_ on reduced cAMP formation would be accelerated and the reverse style curve would shift to the left of the *dashed wine-red curve* of PGD_2_ alone. However, although the effect was weak, DK-PGD_2_ could activate DP receptors by itself, and the formation of cAMP was step-wisely and slightly prolonged even after PGD_2_ had been completely metabolized to DK-PGD_2_. However, if all PGD_2_s were metabolized to 15-d-PGD_2_ followed by 15-d-PGJ_2_, as plotted on the *dashed sky-blue line*, the curve should overlap with that of PGD_2_ alone (*dashed wine-red line*), since these metabolites showed no efficacies and potencies, as presented in Figure 2*A*, that is, they could not bind to DP receptors at least a concentration of 100 nM (data not shown). Conversely, if all PGD_2_s were metabolized to PGJ_2_, the formed cAMP would be sustained and/or even slightly enhanced until PGJ_2_ decreases to around 10^-9^ M (Fig. 3*A*,m), because the concentration–response curve of PGJ_2_, shown in Figure 2*A*, shifted to the left of PGD_2_. Subsequently, with further metabolization of PGJ_2_ to 15-d-PGJ_2_, the formation of cAMP would decrease, and the reverse style curve should be similar to the curve of PGJ_2_ alone, since 15-d-PGJ_2_ would not compete with PGJ_2_ for DP receptors (*blue line*). However, if all PGJ_2_s were further metabolized to Δ^12^-PGJ_2_, this second metabolite could activate DP receptors as a partial agonist, and the formation of cAMP would be step-wisely prolonged after PGJ_2_ had been completely metabolized to Δ^12^-PGJ_2_ and then decrease in reverse increments (*salmon-pink line*) to approximately 10^-10^ nM (Fig. 3*A*,s). In the case of TCF/β-catenin-mediated pathways, as shown in Figure 3*C*, overall tendencies were similar to cAMP formation, presented in Figure 3*B*. However, in the operational area, when 1st-metabolite PGJ_2_ was metabolized to Δ^12^-PGJ_2_, the TCF/β-catenin-mediated pathway decreased faster than that of 15-d-PGJ_2_ (*salmon-pink line*), because Δ^12^-PGJ_2_ retains its affinity for DP receptors as a partial agonist, and so this metabolite would compete with PGJ_2_ so that the effect of PGJ_2_ on the decline in the signaling pathway would be slightly accelerated and the reverse style curve would shift to the left of that of 15-d-PGJ_2_ (*blue line*), which would not compete with PGJ_2_ so that the effect of PGJ_2_ would be retained for slightly longer than that of Δ^12^-PGJ_2_. Of note, there is currently no method to examine the precise timing of metabolism or the exact amounts of metabolites. Therefore, the horizontal axes shown in Figure 3 were the arbitrary timeline, and the actual time span was expected to be very short, for example, 0.9 min or longer, for example, a few hours. Similarly, the vertical axes shown in Figure 3 were arbitrary responses, and the actual amounts of cAMP formed as well as TCF/β-catenin-mediated transcriptional activities may be higher or lower. Nevertheless, the horizontal and vertical scales may fluctuate; however, the PGD_2_-induced trace curves and those of its metabolites may be similar to that shown in Figure 3.

**Figure 4:**
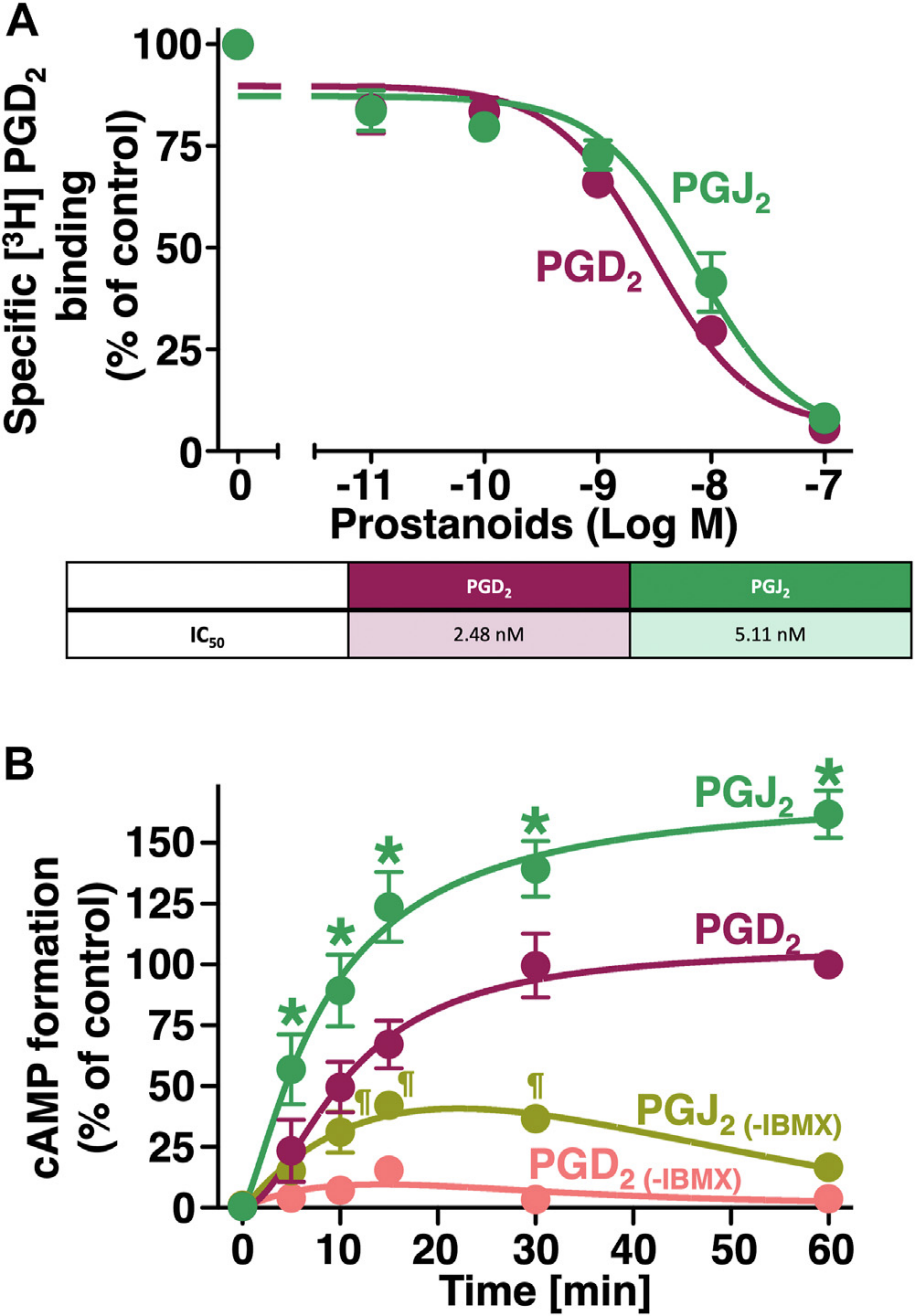
**The competitive whole-cell radioligand binding of [^3^H]PGD_2_ with PGD_2_ or PGJ_2_ and the time-dependent effects of PGD_2_ or PGJ_2_ with or without IBMX pretreatment on cAMP formation in HEK-DP cells**. *A*, HEK-DP cells were trypsinized and resuspended in HEPES buffer, and cell samples were added to the vehicle or indicated concentrations of PGD_2_ or PGJ_2_ for 120 min, followed by washing, and were then assayed for specific binding to [^3^H]PGD_2_. The table shows IC_50_ values obtained from the PGD_2_- or PGJ_2_-competitive [^3^H]PGD_2_ radioligand whole-cell binding assay in HEK-DP cells. *B*, HEK-DP cells were cultured, pretreated with or without 0.1 mg/ml of IBMX for 25 min, and then stimulated with 0.3 nM PGD_2_ or PGJ_2_ for the indicated times. Data are normalized to each vehicle-treated control (*A*) or 0.3 nM PGD_2_-induced amounts of cAMP pretreated with IBMX at 60 min (*B*) as 100% and are the mean ± SD (error bars) of at least three independent experiments, each performed in duplicate. ∗*p* < 0.05: analysis of variance for PGJ_2_ with IBMX significantly higher from the corresponding timepoints of PGD_2_ with IBMX. ¶*p* < 0.05: analysis of variance for PGJ_2_ without IBMX significantly higher from the corresponding timepoints of PGD_2_ without IBMX. HEK-DP cells, human embryonic kidney-293 cell stably expressing human DP receptors; IBMX, 3-isobutyl-1-methylxanthine; PGD_2_, prostaglandin D_2_; PGJ_2_, prostaglandin J_2_.

According to the results obtained in Figure 3, the signaling pathways activated by PGD_2_ would be continuously, sustainingly, and/or even enhanced only when PGD_2_ is metabolized to PGJ_2_. This may be due to the potency of PGJ_2_ for DP receptors, which was approximately 10 times stronger than PGD_2_, especially in cAMP formation, as shown in Figure 2*A*. Therefore, to examine the affinities of PGJ_2_ and PGD_2_ for DP receptors, the competitive receptor binding [^3^H]PGD_2_ assay was performed. As shown in Figure 4*A*, although PGD_2_ showed slightly, but not significantly, higher affinity, PGD_2_ and PGJ_2_ similarly caused the concentration-dependent inhibition of [^3^H]PGD_2_ binding to DP receptors with similar IC_50_ values of 2.48 and 5.11 nM, respectively. Since the binding curves and IC_50_ values of PGD_2_ and PGJ_2_ for DP receptors were similar, the marked potency of PGJ_2_ may not attributed to high affinity for DP receptors.

Besides the affinity, another possible explanation for the increase in cAMP is an increase in adenylyl cyclase (AC) activity and/or decrease in phosphodiesterase (PDE) activity. Therefore, to examine the possibilities, the time-dependent formation of cAMP induced by PGJ_2_ or PGD_2_ was measured under conditions with or without a PDE inhibiter, 3-isobutyl-1-methylxanthine (IBMX). With IBMX pretreatment, PDE should be inhibited so that total cAMP amounts would solely reflect the activation of AC. Alternatively, without IBMX pretreatment, formed cAMP would quickly degrade to AMP so that total cAMP amounts would reflect AC activity with PDE activity. Thus, HEK-DP cells were pretreated with or without IBMX and then treated with 0.3 nM of PGJ_2_ or PGD_2_ for the indicated times, since 0.3 nM was the EC_50_ value of PGD_2_, as shown in Figure 2*A*. Figure 4*B* shows that in the presence of IBMX-pretreated HEK-DP cells, PGJ_2_ could form significantly more cAMP from 5 to 60 min compared with PGD_2_. Similarly, without IBMX pretreatment, because any cAMP formed would be degraded, amounts of cAMP formed were clearly lower than with IBMX. However, PGJ_2_ could also significantly form more cAMP from 10 to 30 min compared with PGD_2_. Therefore, PGJ_2_ may not activate PDE to the level of PGD_2_, or PGD_2_ may not activate AC to the level of PGJ_2_, indicating that PGJ_2_ relatively activates AC over PDE through DP receptors more strongly than PGD_2_.

**Figure 5:**
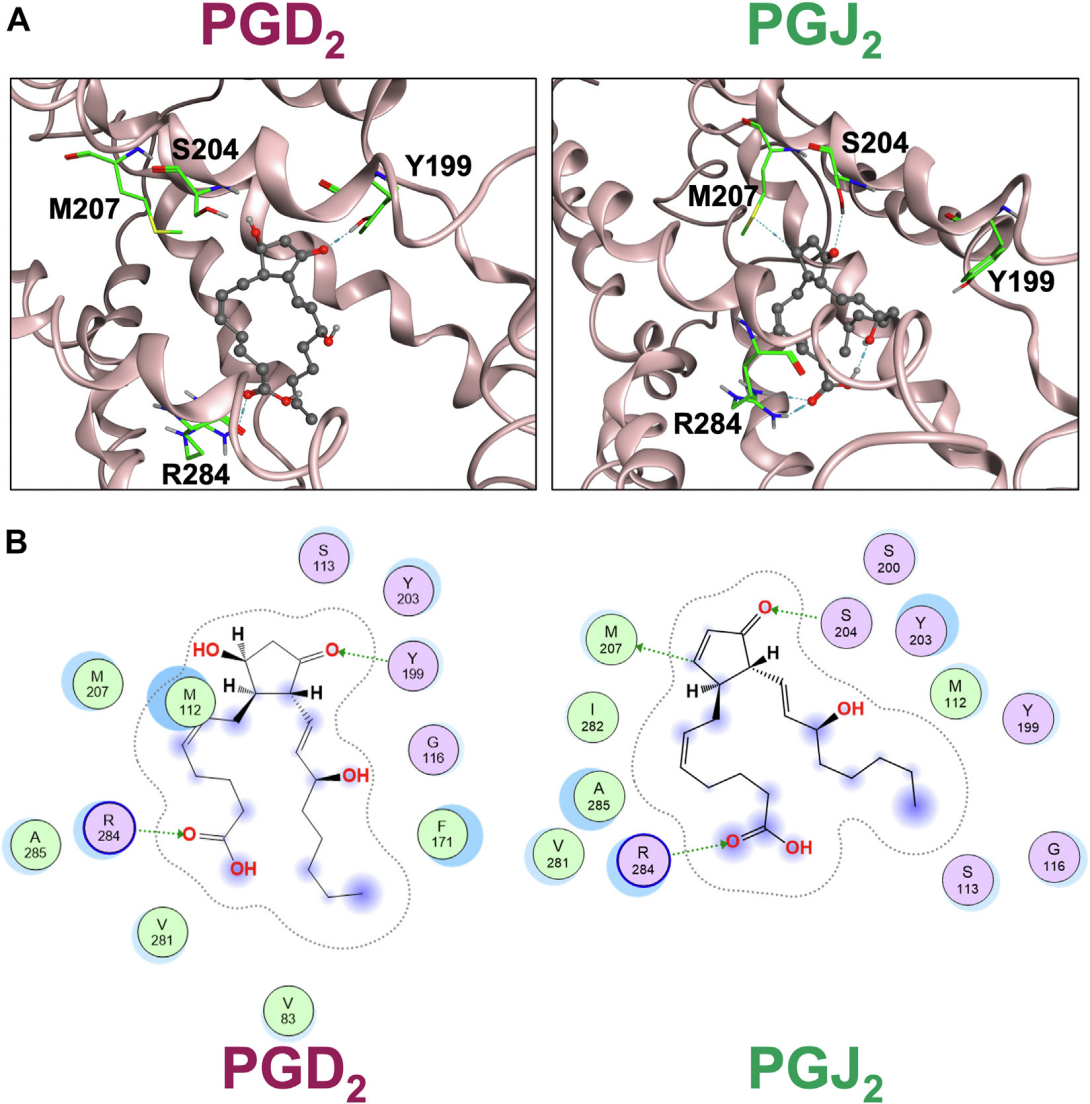
**Binding models of DP receptors with either PGD_2_ or PGJ**_**2.**_*A*, molecular interactions with PGD_2_ (*left panel*) or PGJ_2_ (*right panel*) binding cavities of the receptors. *Dashed lines*: hydrogen bonds. *B*, schematic representations of hydrogen bondings between DP receptors and PGD_2_ (*left panel*) or PGJ_2_ (*right panel*). *Dashed arrows*: hydrogen bonds from acceptors to donors. *Blue color circles* around the ligands and residues indicate contacts with the solvent. Each hydrogen bond between ligand and receptor was estimated by Molecular Operating Environment software calculation. DP, D-type prostanoid; PGD_2_, prostaglandin D_2_; PGJ_2_, prostaglandin J_2_.

Next, to further understand the reasons why PGJ_2_ showed stronger potency than PGD_2_ for DP receptors, *in silico* simulation was performed to estimate the interaction of PGD_2_ or PGJ_2_ with DP receptors. As depicted in Figure 5*A*, both PGD_2_ and PGJ_2_ were estimated to bind to the cavities of the DP receptors but differently. As estimated in Figure 5*B*, PGD_2_ formed hydrogen bonds with arginine (R) 284 of the receptor with the first position of its carboxyl functional group, and with tyrosine (Y) 199 of the DP receptor with the 11th position of carbonyl in its cyclopentane ring. PGJ_2_ also formed a hydrogen bond with R284 with the first position of its carboxyl functional group in the same way as PGD_2_; however, the 11th position of carbonyl in its cyclopentene ring formed a hydrogen bond with serine (S) 204, instead of Y199, in the DP receptor. Moreover, an additional hydrogen bond was formed between the ninth position of PGJ_2_ and methionine (M) 207 of the DP receptor, as shown in Figure 5*B*. Noteworthy, our simulation estimated a unique, nonclassical C-H•••S hydrogen bond, which can be formed and reported previously (24). Thus, since M207 is located in the fifth transmembrane of DP receptors (7), the additional hydrogen bond formed by PGJ_2_ may change the conformation of receptors to induce more activity in AC or less activity in PDE than PGD_2_.

**Figure 6:**
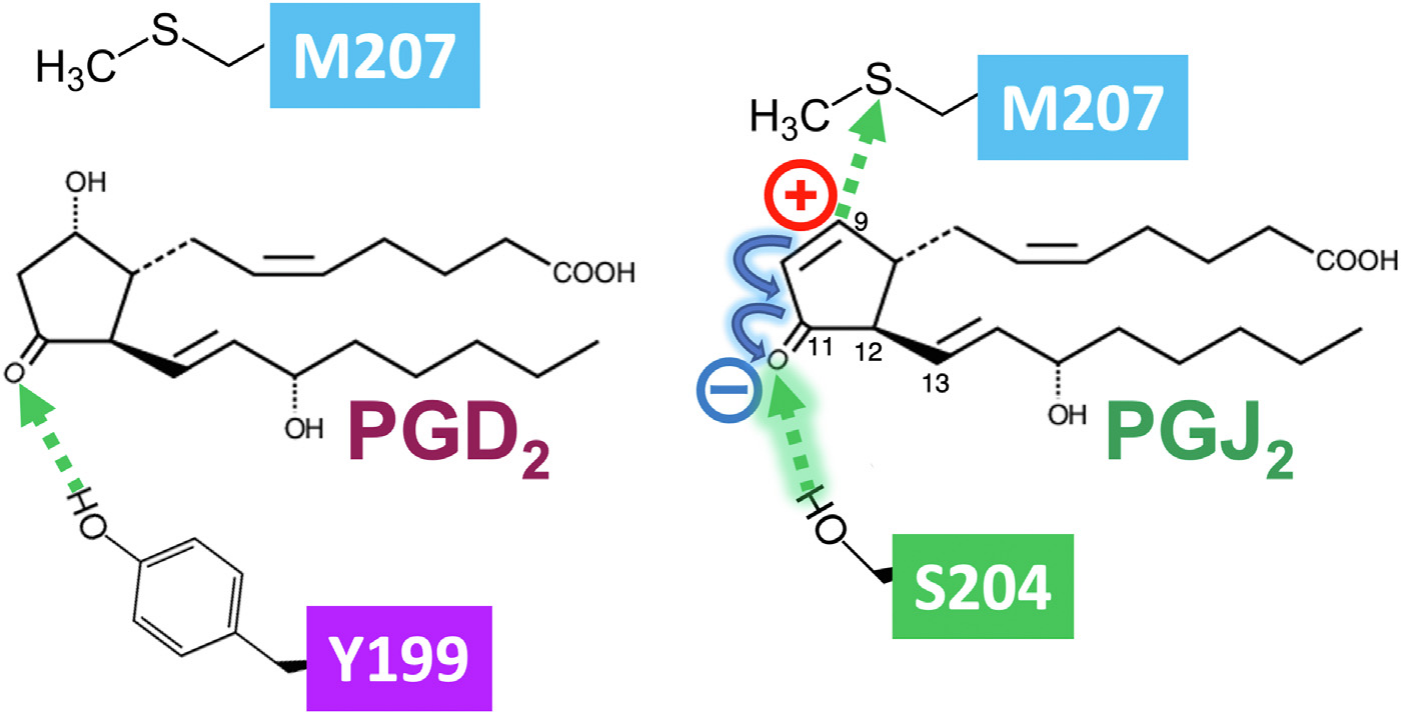
**Schematic hypothesis on interactions between PGD_2_ or PGJ_2_ and DP receptors**. PGD_2_ bound to and formed a hydrogen bond with Y199 of the DP receptor with the 11th position of carbonyl in its cyclopentane ring. Besides an additional hydrogen bond between the ninth position of PGJ_2_ and M207 of the DP receptor, the stronger hydrogen bond would be formed between the 11th position of carbonyl in its cyclopentene ring and S204 of the DP receptor; because PGJ_2_ has an electrophilic α,β-unsaturated carbonyl group in its cyclopentene ring, the carbon at the ninth position is considered electrophilic and chemically reactive. The positively charged carbon at the ninth position of PGJ_2_ would make the carbonyl in the cyclopentene ring more negatively charged by electronic flow than PGD_2_. DP, D-type prostanoid; PGD_2_, prostaglandin D_2_; PGJ_2_, prostaglandin J_2_.

Furthermore, besides the additional hydrogen bond between the ninth position of PGJ_2_ and M207 of the DP receptor, the stronger hydrogen bond would be formed between the 11th position of carbonyl in its cyclopentene ring and S204 of the DP receptor. Thus, as shown in Figure 6, because PGJ_2_ has an electrophilic α,β-unsaturated carbonyl group in its cyclopentene ring, the carbon at the ninth position is considered electrophilic and chemically reactive. Therefore, the positive charge of carbon at the ninth position of PGJ_2_ would make the carbonyl in the cyclopentene ring more negatively charged by electronic flow than PGD_2_, as presented in Figure 6. Theoretically, similar interaction may also be formed between Δ^12^-PGJ_2_ and/or 15-d-PGJ_2_ with DP receptors. As shown in Figure 2, since Δ^12^-PGJ_2_, but not 15-d-PGJ_2_, showed partial agonistic effects on cAMP formation and TCF/β-catenin transcriptional activity. Therefore, there is a possibility that Δ^12^-PGJ_2_ could form similar interactions with DP receptors. However, not only 15-d-PGJ_2_ but also Δ^12^-PGJ_2_ metabolites have a double bond at the 12th position. This double bond would become an additional conjugated double bond, and the hydrogen bond between carbonyl in the cyclopentene ring and S204 of the DP receptor would not be so prominent as in PGJ_2_, since electronic flow would further move to the 12th double bond. Of note, as described in the *Introduction* section, Δ^12^-PGJ_2_ and 15-d-PGJ_2_ have additional reactive carbons at the 13th position of both prostanoids; however, these carbons may not contribute to the activation of DP receptors.

**Figure 7:**
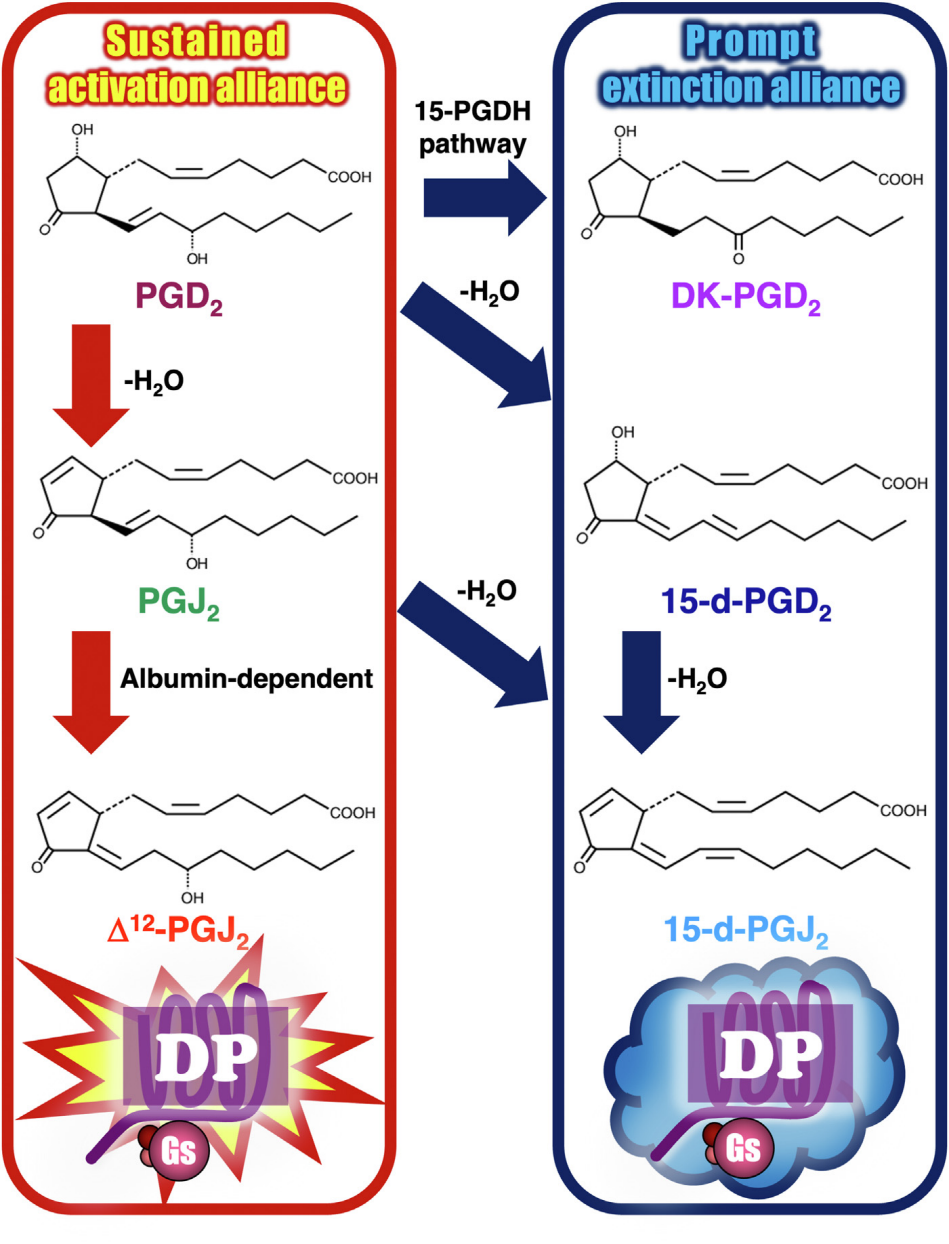
**The sustained activation alliance and prompt extinction alliance of PGD_2_ and its metabolites.** When PGD_2_ is metabolized to DK-PGD_2_ or the line of 15-d-PGD_2_ to 15-d-PGJ_2_, a prompt extinction alliance, activities of DP receptors stimulated by PGD_2_ would be promptly terminated. If PGD_2_ is metabolized to the line of PGJ_2_ to Δ^12^-PGJ_2_, a sustained activation alliance, activities of DP receptors stimulated by PGD_2_ would be sustained or even enhanced. Depending on which metabolic line would be activated, the physiological functions of PGD_2_-stimulated DP receptors would be markedly changed. DK-PGD_2_, 13,14-dihydro-15-keto PGD_2_; 15-d-PGD_2_, 15-deoxy-Δ^12,14^-PGD_2_; 15-d-PGJ_2_, 15-deoxy-Δ^12,14^-PGJ_2_; 15-PGDH, 15-hydroxyprostaglandin dehydrogenase; DP, D-type prosatnoid; PGD_2_, prostaglandin D_2_; PGJ_2_, prostaglandin J_2_.

As described earlier, PGD_2_ will be primarily metabolized to DK-PGD_2_, 15-d-PGD_2_, and PGJ_2_, as shown in Figure 1. Unlike the 15-keto-PGE_2_ on EP2 receptors, as described in the *Introduction* section (20), enzymatically metabolized DK-PGD_2_ showed only slight activities in terms of DP receptor–mediated signaling pathways, as presented in Figure 2. Therefore, 15-hydroxyprostaglandin dehydrogenase pathway may promptly metabolize to DK-PGD_2_, and this metabolite would be more likely to step-wisely terminate the activities stimulated by PGD_2_ of DP receptors. In the case of nonenzymatically metabolized 15-d-PGD_2_ and PGJ_2_, these prostanoids induced totally opposite functions from DP receptors even though they are both spontaneous dehydration products of PGD_2_. Thus, at least up to 100 nM, 15-d-PGD_2_ may be a completely inactive metabolite, whereas PGJ_2_ may be a more potent biased agonist in terms of the Gs-protein/cAMP-mediated pathway than PGD_2_, at least for DP receptors. As simulated in Figure 3, when PGD_2_ was metabolized to 15-d-PGD_2_, this prostanoid immediately terminated the activities promoted by DP receptors, being similar to DK-PGD_2_. However, with the metabolization of PGD_2_ to PGJ_2_, this prostanoid took over/out competed or even enhanced the activities stimulated by PGD_2_ and succeeded in continuous and sustained DP receptor activation. Of interest, with further metabolization of PGJ_2_ to 15-d-PGJ_2_, this prostanoid terminated PGJ_2_-evoked sustained activities, but on even further metabolization of PGJ_2_ to Δ^12^-PGJ_2_, this prostanoid promoted the sustained activities, then gradually reduced and terminated DP receptor–mediated activities. Therefore, when PGD_2_ is metabolized to DK-PGD_2_ or the line of 15-d-PGD_2_ to 15-d-PGJ_2_, a prompt extinction alliance, as depicted in Figure 7, activities of DP receptors stimulated by PGD_2_ would be promptly terminated. Conversely, if PGD_2_ is metabolized to the line of PGJ_2_ to Δ^12^-PGJ_2_, a sustained activation alliance, activities of DP receptors stimulated by PGD_2_ would be sustained or even enhanced, and then smoothly terminated. If PGD_2_ is metabolized to the line of PGJ_2_ to 15-d-PGJ_2_, the activities of DP receptors would be prolonged until PGJ_2_ has been completely metabolized since 15-d-PGJ_2_ barely activates DP receptors because of its little efficacy, so termination would be faster when compared with the sustained line of PGJ_2_ to Δ^12^-PGJ_2_. Thus, dependent on which metabolic line would be activated, the physiological functions of PGD_2_-stimulated DP receptors would markedly change. In addition, other than PGJ_2_, PGD_2_ metabolites did not compete with [^3^H]PGD_2_ in the DP receptor binding assay, at least to a concentration of 100 nM; therefore, they may not exert the antagonistic effects of PGD_2_ on DP receptors (data not shown).

As shown in Figures 1 and 6, although it is considered as spontaneous nonenzymatic dehydration, isomerlization to Δ^12^-PGJ_2_ from PGJ_2_ is well known as albumin dependent (3, 9, 11, 16). Indeed, without albumin, PGJ_2_ would be metabolized to 15-d-PGJ_2_, but not Δ^12^-PGJ_2_, suggesting that albumin would be highly inhibitory of 15-d-PGJ_2_ production (9). Thus, the line of PGJ_2_ to Δ^12^-PGJ_2_, a sustained activation alliance, plausibly functions only when PGD_2_ is released extracellularly where serum albumin exists. However, the line of 15-d-PGD_2_ to 15-d-PGJ_2_, a prompt extinction alliance, as well as the line of PGJ_2_ to 15-d-PGJ_2_ would function intracellularly where no serum albumin is available. This may be reasonable since 15-d-PGJ_2_ showed little effect on DP receptors, but it has been extensively reported to activate PPARγ as a potent agonist (11, 17, 18). Therefore, when PGD_2_ is released into the blood stream, this prostanoid would stimulate DP receptors, and activated DP receptors would be further and continuously activated by PGJ_2_ followed by Δ^12^-PGJ_2_ to sustain the DP receptor/cAMP-mediated signaling pathway.

Intriguingly, PGJ_2_ has been reported to exhibit pleiotropic characters (11), since it exerts its functions through receptor-dependent as well as receptor-independent mechanisms (11). Also, there are three receptors that can be activated by PGJ_2_: DP receptors, CRTH2 receptors, and PPARs (11). With respect to receptor-independent mechanisms, the carbon at the ninth position can form covalent Michael adducts with cellular proteins, including glutathione (11, 17, 18). However, the functions of PGJ_2_ can be largely and roughly divided into two: anti-inflammatory or proinflammatory functions (11). Thus, when PGJ_2_ acts on CRTH2 receptors or in receptor-independent ways, it would show proinflammatory functions. However, as shown and discussed previously, when PGJ_2_ acts on DP receptors, it may enhance and prolong anti-inflammatory functions as a primary metabolite of PGD_2_ along with the secondary metabolite, Δ^12^-PGJ_2_, in an environment containing albumin (3, 9, 11, 16). Moreover, when PGJ_2_ is intracellularly metabolized to 15-d-PGJ_2_, another secondary metabolite, this prostanoid would also evoke anti-inflammatory functions, not *via* DP receptors, but as a potent agonist for PPARs (11, 17, 18).

## Conclusions

Among the five metabolites of PGD_2_, we here show that PGJ_2_ is the most potent metabolite of DP receptors, particularly in the cAMP signaling pathway. Many mechanisms may explain outcome differences among the ligands, such as different rates of desensitization of the DP receptors. However, according to the simulated results, it may be originally caused by ligand-dependent conformational changes in DP receptors *via* ligand-specific hydrogen bond formations, that is, PGJ_2_ forms an extra hydrogen bond, and/or make the hydrogen bond stronger between the carbonyl in the cyclopentene ring and S204 by the positive charge of the carbon at ninth position of PGJ_2_ with DP receptors than PGD_2_, as shown in Figure 5.

It is well known that PGD_2_ is readily metabolized so that its half-life in blood has been reported as 0.9 min (19) or 30 min (16). Therefore, anti-inflammatory effects of PGD_2_ are taken over and/or even enhanced by its metabolite PGJ_2_. Indeed, it was reported that collagen-induced platelet aggregation was inhibited by PGD_2_, and PGJ_2_ compensated for the effects of PGD_2_ after PGD_2_ was degraded (16). However, the potency of inhibiting platelet aggregation by PGJ_2_ was 13 times lower than that of PGD_2_, and it is possible to consider that PGJ_2_ may not only activate DP receptors expressed on platelets but other receptors/factors such as T-type prostanoid receptors and/or PPARγ, or it was quickly metabolized to Δ^12^-PGJ_2_ by the albumin in the preparation buffer. Alternatively, the weaker effects of PGJ_2_ could be due to the biased activities of this prostanoid. Thus, the higher activity of cAMP formation by PGJ_2_ on DP receptors would affect the downstream signaling pathway of platelets, resulting in the weaker physiological functions of their aggregation. Definitely, it is necessary to validate the effects of PGD_2_ and its metabolites on other PGD_2_ receptors, CRTH2 receptors, as well as PPARs whether they would also show similar alliances on activation or inhibition. It is also necessary to examine this using actual immune cell lines to confirm the findings. Furthermore, confirmation of the results of simulations is necessary in future experiments. Nevertheless, our cell-based results, calculations, and simulations showed that PGJ_2_ may not be just a weak intermediate metabolite of PGD_2_, but a standout biased mediator of the Gs-protein-mediated signaling pathway than β-arrestin-mediated TCF/β-catenin-mediated pathway, which can provoke more potent and prolonged activities of DP receptors as a biased ligand elicited by its precursor mediator, PGD_2_, that is, for maintaining homeostasis to actively resolve the inflammatory reaction.

In contrast to the least abundant DP receptors, their primary ligand PGD_2_ is acknowledged as a major prostanoid in most tissues. Although PGD_2_ is well known to be readily metabolized, its metabolites are considered more stable than their precursor. Therefore, variety of the physiological functions from DP receptor activation may not be defined by PGD_2_ itself, but plausibly or possibly, by its metabolites.

## Experimental procedures

### Cell culture and materials

HEK-DP cells were generated and provided as described previously (25) that were cultured in 10% fetal bovine serum (Thermo Fisher Scientific), 250 μg/ml geneticin (Phyto Technology Laboratories), 200 μg/ml hygromycin B (Enzo Life Sciences), and 100 μg/ml gentamicin (Thermo Fisher Scientific) at 37 °C in Dulbecco's modified Eagle's medium (DMEM; Nacalai Tesque) under mycoplasma-free condition. HEK-DP cells showed the maximal level of specific [^3^H]PGD_2_ binding that is 20-fold or more the amount of specific [^3^H]PGD_2_ binding to untransfected parental HEK-293 cells (HEK, −0.161 ± 1.46 fmol/mg protein; HEK-DP, 21.7 ± 5.69 fmol/mg protein). PGD_2_, DK-PGD_2_, PGJ_2_, 15-d-PGD_2_, Δ^12^-PGJ_2_, and 15-d-PGJ_2_ were purchased from Cayman Chemical. All materials were obtained from FUJIFILM Wako Pure Chemical Corp unless otherwise stated.

### cAMP assay

HEK-DP cells were cultured in 6-well plates and, prior to the experiments, the medium was switched from DMEM to Opti-MEM (Thermo Fisher Scientific) containing 250 μg/ml geneticin, 200 μg/ml hygromycin B, and 100 μg/ml gentamicin for 16 h. Cells were then pretreated with or without 0.1 mg/ml of IBMX (Sigma–Aldrich) for 25 min followed by treatment with vehicle (0.1% Me_2_SO) or the indicated concentrations of PGD_2_ or its metabolites for 60 min, or for the indicated times at 37 °C. Three hundred microliters of TE buffer (50 mM Tris–HCl, 4 mM EDTA [pH 7.5]) was added, and cells were scraped and transferred to microcentrifuge tubes. Samples were boiled for 8 min, placed on ice, and then centrifuged for 1 min at 16,000*g*. Between 5 and 25 μl of the supernatants representing approximately 10^4^ cells was added to 50 μl of TE buffer containing [^3^H]cAMP (PerkinElmer Life Science) and then to 100 μl of 0.06 mg/ml PKA (Sigma–Aldrich; #P5511). The mixture was vortexed and incubated on ice for 120 min. Samples were added to 100 μl of TE buffer containing 2% of bovine serum albumin and 26 mg/ml of powdered activated charcoal (Sigma–Aldrich; #C7606). After vortexing and centrifugation at 16,000*g* for 5 min, 200 μl of the supernatants were transferred to vials for liquid scintillation. The amount of cAMP that had formed was calculated from a standard curve prepared using nonradiolabeled cAMP, as described previously (20). When performing the cAMP assay stimulated with each PGD_2_ or PGD_2_ metabolite, an extra assay, which was stimulated with 100 nM PGD_2_ as a control, was also conducted, and all data were normalized using each result of 100 nM PGD_2_ as the standard (100%).

### TCF/**β**-catenin-mediated luciferase reporter assay

HEK-DP cells were cultured in 6-well plates and, prior to the experiments, medium was switched to Opti-MEM for 16 h with antibiotics, as stated previously. Cells were transiently transfected using HyliMax reagent (Dojindo) with 400 ng/well of either TOP flash or FOP flash reporter plasmids and 2 ng/well of pRL-CMV plasmid (Promega), as described previously (20). Cells were treated with either vehicle (0.1% Me_2_SO) or the indicated concentrations of PGD_2_ or its metabolites for 16 h. Cells were then lysed and assayed using a dual luciferase reporter assay system (Promega) according to the manufacturer's instructions with TECAN infinite M200 (TECAN). Data were normalized by calculating the ratios of firefly luciferase values to corresponding renilla luciferase scores and corrected for background activity by subtracting FOP flash scores from the corresponding TOP flash values, as described previously (20).

### Black/Leff operational model

The estimated affinities (K_A_ values of DK-PGD_2_ and Δ^12^-PGJ_2_) and Tau (τ) values were determined by GraphPad Prism software (version 9.3.0; GraphPad Software, Inc). The equation "operational model-partial agonist" was performed using the results presented in Figure 2, as reported previously (20). Effectmax values used the PGD_2_-evoked maximal effects of cAMP formation and TCF-mediated signaling presented in Figure 2. The basal level was 0 (Fig. 2*A*; cAMP) or 1 (Fig. 2*B*, TCF), and Hill slopes were specified as 1. In the case of 15-d-PGD_2_ and 15-d-PGJ_2_, K_A_ values were set as 1 and τ values were set as 0 since neither prostanoid was evoked in signaling pathways, as shown in Figure 2. For partial agonists, DK-PGD_2_ and Δ^12^-PGJ_2_, the transduction coefficient (log R, R = (τ/K_A_)), the system/surrounding environment–independent parameter considering affinity and efficacy of the agonist (20, 21), was obtained from K_A_ and τ calculated by the Black/Leff operational model. Since PGD_2_ and PGJ_2_ acted as full agonists, K_A_ values of PGD_2_ and PGJ_2_ used IC_50_ values of the binding assay as K_A_' values, shown in Figure 4*A*. For PGD_2_ and PGJ_2_, Tau' (τ′), the predicted value of τ, was obtained from τ' = K_A_' x R. The concentration of PGD_2_ was simulated to increase from 0 to 10^-7^ M and then decrease to 0, whereas the concentration of 1st-metabolites of PGD_2_ (DK-PGD_2_, 15-d-PGD_2_, and PGJ_2_) was simulated assuming a 1:1 conversion ratio of PGD_2_. Then, the concentration of each 1st-metabolite was simulated to decrease from 10^-7^ M to 0, whereas the concentration of 2nd-metabolites (15-d-PGD_2_ was metabolized to 15-d-PGJ_2_; PGJ_2_ was metabolized to 15-d-PGJ_2_ or Δ^12^-PGJ_2_) was simulated assuming 1:1 conversion of 1st-metabolites and then a decrease to 0. The total responses evoked by the precursor prostanoid and its metabolites were calculated using the formula below (23):

$$\text{Response}=\frac{\frac{\left[A1\right]}{KA1}\;\times \;Tau1\;+\;\frac{\left[A2\right]}{KA2}\;\times \;Tau2}{\frac{\left[A1\right]}{KA1}\;\times \;\left(Tau1\;+\;1\right)\;+\;\frac{\left[A2\right]}{KA2}\;\times \;\left(Tau2\;+\;1\right)\;+\;1}\;\times \;EffectMax$$

KA1: K_A_' value of PGD_2_, alternatively K_A_' value of PGJ_2_ or K_A_ values of 1st-metabolites, KA2: K_A_' value of PGJ_2_ or K_A_ values of 1st-metabolites, alternatively K_A_ values of 2nd-metabolites.

[A1]: the concentration of PGD_2_, alternatively the concentrations of 1st-metabolites as defined previously. [A2]: the concentrations of 1st-metabolites, alternatively the concentrations of 2nd-metabolites as defined previously Tau1: τ′ value of PGD_2_, alternatively τ′ value of PGJ_2,_ or τ values of 1st-metabolites. Tau2: τ′ value of PGJ_2_ or τ values of 1st-metabolites, alternatively τ values of 2nd-metabolites.

The amounts of cAMP formed and activated TCF/β-catenin-mediated transcription evoked by PGD_2_ at 0 M (a) to 10^-7^ M (f) and by 2nd-metabolites at 10^-7^ (p) to 0 M (u) shown in Figure 3*A* were plotted as curves.

### Binding assay

The medium of HEK-DP cells cultured in 10-cm dishes was switched from DMEM containing 10% fetal bovine serum to Opti-MEM at 37 °C for 16 h containing antibiotics, as stated previously. Cells were trypsinized and then resuspended at 1.0 x 10^6^ cells/sample in 100 μl of ice-cold 10 mM HEPES buffer (pH 7.4) containing 1 mM EDTA and 10 mM MnCl_2_ (Sigma–Aldrich). A total of 0.8 nM [^3^H]PGD_2_ (Perkin–Elmer) was used for the binding assay, as shown previously (25) with increased concentrations of PGD_2_ or PGJ_2_. Samples were incubated for 2 h at 4 ^o^C, and then the assay was terminated by filtration through Whatman GF/C glass filters (Whatman). Filters were then washed three to five times with ice-cold HEPES buffer, and radioactivity was measured by liquid scintillation counting (20).

### Molecular modeling simulation of protein–ligand complexes

Constructions of the three-dimensional structures of human DP receptors and docking simulations of PGD_2_ or PGJ_2_ to DP receptors were performed with the Molecular Operating Environment (MOE, version 2022.02, Chemical Computing Group, Inc) based on the Protein Data Bank entry 4GRV.

## Data availability

Data for constructions of the three-dimensional structures of DP receptors and docking simulations of PGD_2_ or PGJ_2_ to DP receptors were based on the Protein Data Bank entry 4GRV (www.rcsb.org/structure/4GRV). The rest of data are contained within the article.

## Conflict of interest

The authors declare that they have no conflicts of interest with the contents of this article.
